# Ichthyosis uteri complicated by endometrial adenocarcinoma with transitional cell differentiation: A case report

**DOI:** 10.1097/MD.0000000000038792

**Published:** 2024-07-12

**Authors:** Liya Ding, Wangwang Liu, Hui Li, Dingpin Huang, Yang Chen, Huimin An

**Affiliations:** a Department of Pathology, Sir Run Run Shaw Hospital, Zhejiang University School of Medicine, Hangzhou 310000, China; b Department of Radiology, Sir Run Run Shaw Hospital, Zhejiang University School of Medicine, Hangzhou 310000, China; c Department of Medical Ultrasonics, Sir Run Run Shaw Hospital, Zhejiang University School of Medicine, Hangzhou, Zhejiang, China

**Keywords:** endometrioid adenocarcinoma, gene mutation, ichthyosis uteri, metaplasia, transitional cell differentiation

## Abstract

**Rationale:**

Ichthyosis uteri is a rare pathological condition characterized by the replacement of the endometrial lining by stratified squamous epithelium. Yet its occurrence with endometrial adenocarcinoma is very rare.

**Patient concerns:**

A 68-year-old woman has been experiencing sporadic, minor vaginal hemorrhages for a few months. The gynecological evaluation revealed a uterine enlargement and imaging demonstrated an irregular mass within the uterus.

**Diagnosis:**

Endometrial adenocarcinoma with transitional cell differentiation; ichthyosis uteri with dysplasia.

**Interventions:**

Radical hysterectomy with pelvic lymphadenectomy was performed followed by postoperative radiotherapy.

**Outcomes:**

Postoperative follow-up at 8 months showed a favorable outcome without signs of recurrence and metastasis.

**Lessons:**

Adequate pathological sampling is crucial to identifying the accompanying lesions of ichthyosis uteri. Finding molecular alterations in various pathological morphologies is important to understand the evolution of disease.

## 1. Introduction

Endometrioid carcinoma (ECa) is the most common gynecological malignancy within developed nations.^[1]^ Epithelial metaplasia refers to the transformation of the epithelial lining of the uterine cavity from its usual endometrial phenotype to other subtypes. Squamous, morular, and mucinous differentiation is commonly associated with ECa, but the occurrence of transitional cell differentiation (TCD) remains exceedingly rare.^[2]^ Ichthyosis uteri is also a rare condition describing the entire endometrium surface replaced by stratified squamous epithelium, till now fewer than 20 cases have been reported. Since isolated ichthyosis typically exhibits no symptoms, it is unintentionally found when endometrial samples are obtained for other purposes. It has been shown that ichthyosis uteri can coexist with endometrial squamous cell carcinoma^[3]^ or endometrial adenocarcinoma.^[4]^ We investigate the usual gene mutations of this case to contribute to the limited understanding of this disease, and we provide an explanation of the origins of the varied patterns of differentiation based on mutational signatures.

## 2. Case summary

**Figure 1.**
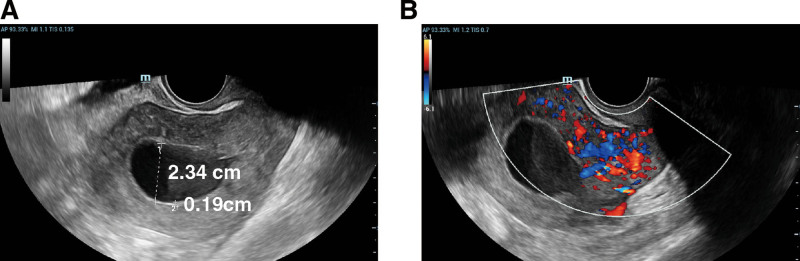
The preoperative transvaginal ultrasound examination of the uterus and bilateral adnexa. (A) Separation of the uterine cavity line; (B) increased echogenicity in the middle-lower segment of the uterine muscle layer, with abundant internal vascularity.

**Figure 2.**
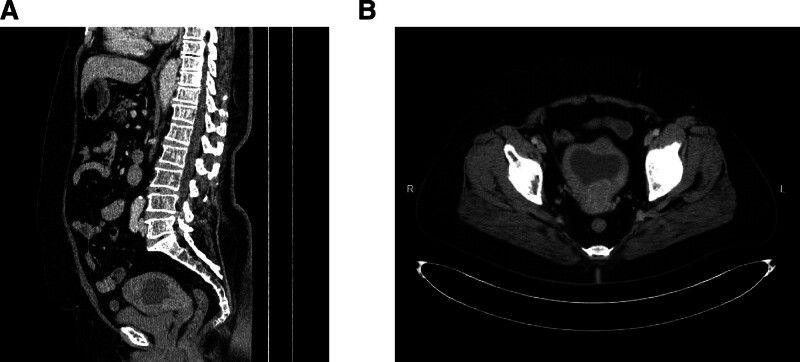
Preoperative contrast-enhanced CT of the entire abdomen reveals an irregular mass in the cervical canal, with indistinct borders and relatively uniform enhancement: (A) sagittal, (B) axial.

A 68-year-old Chinese woman, gravida 4 para 3, was admitted to the gynecology department with a chief complaint of vaginal discharge for 3 months. She admitted to being menopausal for 16 years and denied hormone replacement. She had been diagnosed with hypertension and diabetes and was taking medication to control both conditions. Gynecological examination showed a uterine enlargement. Uterine ultrasound revealed that the uterine cavity was widened by 2.34 cm, with a single-layer endometrial thickness of 0.19 cm (Fig. 1A). Enhanced echogenicity was observed in the middle-lower uterine myometrium with abundant vascularity (Fig. 1B). Abdomen-enhanced CT revealed an irregular mass measuring 37 × 30 mm in the internal cervical canal. It showed moderate but homogeneous enhancement, with indistinct borders. Enlargement of the uterine cavity with fluid accumulation is noted (Fig. 2). Some serum tumor markers were elevated: CA19-9 was 39.4 U/mL (normal, <34 U/ mL), SCC was 6.26 ng/mL (normal, 0–3 ng/mL), and CA242 was 28.6 IU/mL (normal, 0–20 IU/mL). HPV DNA was not detected. The patient underwent a radical hysterectomy with bilateral adnexectomy and pelvic lymph node dissection.

**Figure 3.**
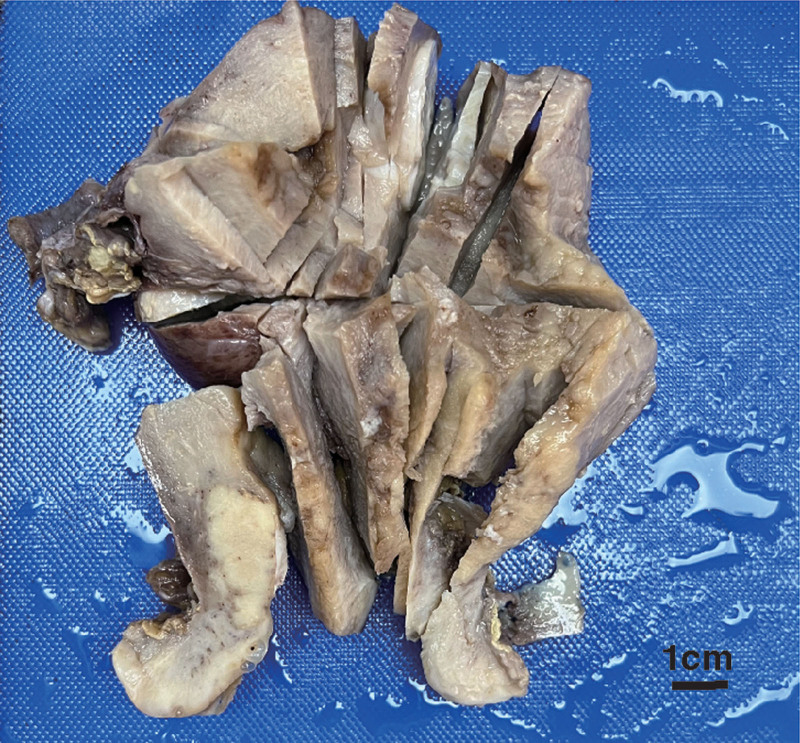
The gross appearance shows a grayish-white mass at the junction of the cervix and the body of the uterus. The surface of the endometrium is rough, with focal areas appearing grayish-white.

Macroscopic examination of the excised specimens showed a tumor measuring 38 × 33 mm mass in the cervico-uterine junction. The surface of the mass is covered with a white lining of the endometrium (Fig. 3). The endometrium of the uterus is extensively rough, with cauliflower-like growth in some areas of the uterine cavity. Two myomas, each measuring 3 cm in diameter, were observed in the uterine corpus.

**Figure 4.**
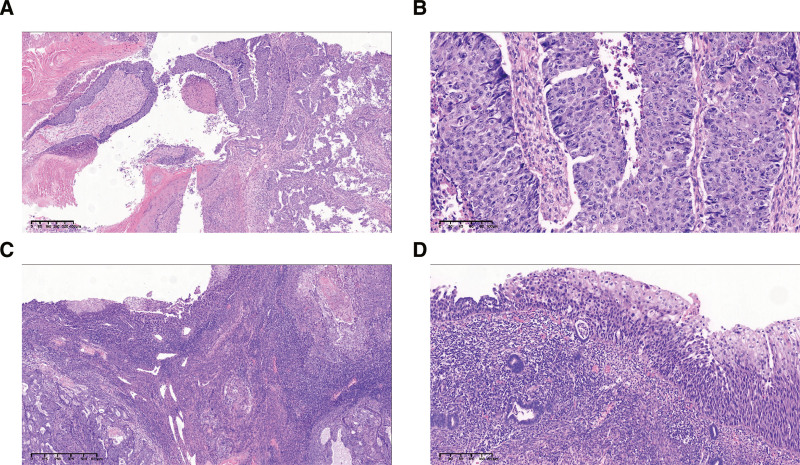
Concurrent endometrial adenocarcinoma with transitional cell differentiation (TCD) in Ichthyosis uteri. (A) Juxtaposed endometrial adenocarcinoma with keratinizing squamous and TCD. (B) Enlarge display of transitional cell differentiation. (C) Stratified squamous epithelium lined on the surface of endometrial adenocarcinoma. (D) Dysplastic squamous epithelium replacing endometrial lining with subepithelial lymphoplasmacytic inflammatory infiltrate.

Histopathologic examination revealed that the tumor predominantly exhibited glandular growth and infiltrated into more than half of the myometrium without involving the cervical glands (Fig. 4A). Hence, the tumor was diagnosed as endometrial adenocarcinoma FIGO grade 2. TCD was found in approximately 1% of the tumors, which were composed of papillary structures with thin fibrovascular cores covered by multilayered polygonal cells (Fig. 4B). The keratinizing squamous epithelium was conspicuous and mixed with the tumor. Stratified squamous epithelial extensively covered the endometrium and the tumor surface, and certain areas displayed diffuse basal and parabasal cell proliferation with nuclear atypia (Fig. 4C). The nuclei are enlarged, pleomorphic, and mitoses are easily visualized (Fig. 4D). A small number of endometrial glands were found under the squamous epithelium, which showed atrophic changes and was surrounded by numerous lymphocytes. Endometrioid carcinoma with foci of TCD and squamous dysplasia in the ichthyosis uteri was diagnosed. There was no evidence of cancer metastasis in the surgically removed lymph nodes. The final pathological staging is pT2N0M0 according to AJCC 8th.

Endometrial adenocarcinoma immunohistochemistry examination showed that ER and PR were positive, CEA-R and CK7 were focally positive, whereas p40, p63, p16, p53, and CK20 were negative. As for the squamous dysplasia area, p40, p63 and p53 were positive, whereas ER, PR, CK7, CK20, and p16 were negative. Ki-67 was 35% positive.

The distinct morphologies underwent mutational analysis, revealing genomic alterations in 4 to 7 genes. All the morphology shared identical mutations in CDKN2A, CTNNB1, and PTEN. The PTEN alteration involved an almost heterozygous missense single-base substitution (SBS), leading to PTEN mutations D92E and R173C. Similarly, CTNNB1 exhibited a heterozygous missense SBS resulting in mutation D32Y. Additionally, CDKN2A gained a frameshift mutation at leucine residue 78, leading to a premature stop codon at position 39 in the protein sequence (p.L78Tfs*39). Compared to the endometrioid adenocarcinoma, the TCD area gained the mutation of PIK3R1 p.K567E and TP53 p.R248Q. The p.R248Q mutation was only found in the TCD area. The keratinizing squamous epithelium exhibited mutations similar to those found in the squamous dysplasia areas. The NFE2L2 p.D77G mutation was present in the endometrioid adenocarcinoma, TCD area, and keratinizing squamous epithelium, but not in the squamous dysplasia. Detailed mutational profiles of the tumors are provided in Table 1.

**Table 1:** Summary of gene alterations and mutation abundance in the endometrioid adenocarcinoma, transitional cell differentiation (TCD), keratinizing squamous epithelium, and squamous dysplasia.

Gene	Description	Endometrioid adenocarcinoma	TCD	Keratinized squamous epithelium	Squamous dysplasia
CDKN2A	p.L78Tfs*39	23.53%	22.14%	21.29%	6.94%
CTNNB1	p.D32Y	31.16%	28.56%	23.21%	12.81%
PIK3R1	p.K567E	/	37.76%	22.56%	9.84%
PTEN	p.D92E	29.53%	31.66%	29.64%	19.10%
PTEN	p.R173C	25.53%	31.55%	19.72%	6.09%
NFE2L2	p.D77G	35.03%	46.83%	15.06%	/
TP53	p.R248Q	/	9.18%	/	/
TP53	p.S367Rfs*12	/	/	10.24%	12.82%
TP53	p.R273H	/	/	3.50%	9.73%
FBXW7	p.R543G	/	/	30.11%	32.44%

Postoperatively, the patient was treated with 10MV-X radiation, targeting the pelvic region. The total prescribed dose is 4500 cGy, divided into 25 treatment fractions, over a treatment duration of 33 days. Eight months after surgery, the patient is alive without any signs of recurrence or metastasis.

## 3. Discussion

Ichthyosis uteri was first reported in 1969 with the characteristic of extensive squamous metaplasia of the endometrium.^[5]^ Endometrial ichthyosis is rare and mostly occurs in elderly postmenopausal women. Historically, abnormal uterine bleeding was usually treated by injecting corrosive substances such as formaldehyde or iodine into the uterine cavity, leading to extensive squamous metaplasia of the endometrium, called endometrial ichthyosis. Subsequent studies identified additional causes, including vitamin A deficiency, endometrial hyperplasia, adenocarcinoma, senile endometrial polyps, chronic endometritis, pyometra, tuberculous endometritis, and radiation therapy.^[6,7]^ Many of the above causes may produce only focal endometrial squamous metaplasia or mulberry-like metaplasia, whereas endometrial ichthyosis manifests as widespread mature keratinizing squamous epithelium. Currently, there are only a few cases that report the cooccurrence of ichthyosis uteri with keratinizing squamous metaplasia,^[8,9]^ squamous cell carcinoma,^[3]^ and endometrioid carcinoma.^[4,10,11]^

Only 2 cases of endometrial cancer with TCD have been reported in the literature.^[12,13]^ The TCD were highly cellular and covered by multilayered polygonal cells with abundant pale eosinophilic cytoplasm, well-defined cell borders, and round to ovoid nuclei. The histogenesis mechanism of TCD of the endometrium is still unknown. The TCD and ECa components in our case had the same mutations in CDKN2A, CTNNB1, PTEN, and NFE2L2. Independently, the TCD acquired the TP53 p.R248Q mutation. In this case, we would prefer that the TCD be oriented from ECa.

The behavior of endometrial ichthyosis is benign and inert, and it is typically unrelated to HPV. It has been confirmed by reports that endometrial ichthyosis can develop into cancer.^[6]^ Additional data suggest that HPV infection may have a role in some cases. which can cause squamous intraepithelial lesions^[8]^ and squamous cell carcinoma.^[14,15]^ Thus, HPV-related cervical lesions have the potential to move upward and include the ichthyosis-like endometrium.^[14]^ There is no HPV infection present in this case and did not have a history of cervical dysplasia. Hence, squamous dyspepsia was developed on endometrial ichthyosis and unrelated to HPV infection.

Squamous metaplastic change is commonly observed in ECa. It has been suggested that p63 expression in ECa indicates squamous differentiation. Morphologically, keratinizing squamous epithelium has partially mixed with ECa. Regarding gene alterations, the keratinizing squamous epithelium shares mutations with squamous dysplasia, excluding NFE2L2, and exhibits identical mutations to those found in ECa. Based on the findings, it is challenging to determine the relationship between squamous dysplasia, ECa, and keratinizing squamous epithelium. The literature indicates that mature keratinizing squamous epithelium can replace a significant portion of the surface endometrium.^[3]^ Therefore, we favor that the keratinizing squamous epithelium is oriented from the endometrial ichthyosis.

## 4. Conclusion

In summary, we reported a rare case exhibiting various morphologies of ECa in ichthyosis uteri. The mutation signature suggested that keratinizing squamous epithelium and squamous dysplasia may have originated from a single precursor lesion, as well as the ECa and TCD components. However, further research is needed to clarify this. Investigations of gene mutations in multiple components of ichthyosis uteri are needed to elucidate the mechanisms involved in the development of these highly different histological components and to identify targets for effective personalized treatments.

## Author contributions

**Conceptualization:** Liya Ding, Wangwang Liu, Huimin An.

**Data curation:** Liya Ding, Wangwang Liu, Hui Li, Dingpin Huang, Yang Chen, Huimin An.

**Formal analysis:** Liya Ding, Hui Li.

**Writing – original draft:** Liya Ding, Huimin An.

**Writing – review & editing:** Liya Ding, Huimin An.

**Validation:** Wangwang Liu, Dingpin Huang.

**Methodology:** Hui Li.

**Resources:** Yang Chen.

**Supervision:** Huimin An.

## References

[1] Siegel RL Miller KD Wagle NS Jemal A. Cancer statistics, 2023. CA Cancer J Clin. 2023;73:17–48. 36633525 10.3322/caac.21763

[2] Malpica A. How to approach the many faces of endometrioid carcinoma. Mod Pathol. 2016;29(Suppl 1):S29–44. 26715172 10.1038/modpathol.2015.142

[3] Puljiz M Marcelic L Danolic D. Ichthyosis uteri associated with squamous cell carcinoma of the endometrium – a case report. Acta Chir Belg. 2023;123:679–81. 35848086 10.1080/00015458.2022.2101332

[4] Bhardwaj N Diwaker P Gogoi P Wadhwa N Mishra K. Ichthyosis uteri associated with endometrial adenocarcinoma: a case report. J Clin Diagn Res. 2017;11:ED24–5. 10.7860/JCDR/2017/27951.10116 PMC5535377 28764184

[5] Sikorowa L. A case of ichthyosis of the uterus in the course of adenocarcinoma of the corpus uteri. Nowotwory. 1969;19:65–70. Przypadek ichtyosis uteri w przebiegu raka gruczolowego trzonu macicy. 5777648

[6] Jain M Kashyap A Biswas R. Primary endometrial squamous cell carcinoma in-situ with extensive icthyosis uteri: a rare case report. J Clin Diagn Res. 2017;11:ED13–4. 10.7860/JCDR/2017/29967.10384 PMC5620786 28969146

[7] Takeuchi K Tsujino T Yabuta M Kitazawa S. A case of primary squamous cell carcinoma of the endometrium associated with extensive “ichthyosis uteri.”. Eur J Gynaecol Oncol. 2012;33:552–4. 23185812

[8] Fadare O. Dysplastic Ichthyosis uteri-like changes of the entire endometrium associated with a squamous cell carcinoma of the uterine cervix. Diagn Pathol. 2006;1:8. 16759364 10.1186/1746-1596-1-8 PMC1475890

[9] Zhang Y Tounsi S Yadav G Masand RP Costales AB. Ichthyosis uteri: a keratinizing squamous metaplasia of the endometrium with premalignant potential. Gynecol Oncol Rep. 2023;46:101165. 36968297 10.1016/j.gore.2023.101165 PMC10033720

[10] Akizawa Y Yamamoto T Kanno T. Two primary cancers: primary squamous cell carcinoma with extensive ichthyosis uteri and cervical endometrioid carcinoma: a case report. Mol Clin Oncol. 2020;13:1–1. 32874574 10.3892/mco.2020.2113 PMC7453379

[11] Bewtra C Xie QM Hunter WJ Jurgensen W. Ichthyosis uteri: a case report and review of the literature. Arch Pathol Lab Med. 2005;129:e124–5. 15859657 10.5858/2005-129-e124-IUACRA

[12] Lininger RA Ashfaq R Albores-Saavedra J Tavassoli FA. Transitional cell carcinoma of the endometrium and endometrial carcinoma with transitional cell differentiation. Cancer. 1997;79:1933–43. 9149020

[13] Marino-Enriquez A Gonzalez-Rocha T Burgos E. Transitional cell carcinoma of the endometrium and endometrial carcinoma with transitional cell differentiation: a clinicopathologic study of 5 cases and review of the literature. Hum Pathol. 2008;39:1606–13. 18620731 10.1016/j.humpath.2008.03.005

[14] Du J Liao X. Superficial spreading squamous cell carcinoma in situ of the cervix involving the endometrium: a rare case presentation and review of literature. Int J Clin Exp Pathol. 2019;12:4162–6. 31933815 PMC6949783

[15] Vijaywargiya K Kachhara N Chahwala Q Ruia A. Carcinoma cervix leading to ichthyosis uteri: a rare case report. J Obstet Gynaecol India. 2021;71:545–9. 34602768 10.1007/s13224-021-01472-3 PMC8440718

